# GS-441524 and molnupiravir are similarly effective for the treatment of cats with feline infectious peritonitis

**DOI:** 10.3389/fvets.2024.1422408

**Published:** 2024-07-18

**Authors:** Okihiro Sase, Tomoko Iwami, Takeru Sasaki, Tadashi Sano

**Affiliations:** ^1^You-Me Animal Hospital, Sakura, Japan; ^2^Obihiro University of Agriculture and Veterinary Medicine, Department of Clinical Veterinary Science, Hokkaido, Japan

**Keywords:** feline infectious peritonitis, GS-441524, molnupiravir, treatment, outcomes

## Abstract

Although not registered for feline infectious peritonitis (FIP) in Japan, nucleoside analogs have shown efficacy and we have been offering them to owners of cats with FIP at our clinic since January 2020. The aim of this study was to investigate outcomes in cats with FIP who received GS-441524 or molnupiravir. Diagnosis of FIP was based on clinical signs, laboratory test results, and the presence of feline coronavirus RNA in blood or effusion aspirate. After providing verbal and written information, owners of cats with a presumptive diagnosis of FIP with a were offered antiviral treatment with commercially sourced GS-441524 from June 2020, and either GS-441524 or compounded molnupiravir from January 2022. Dosing was 12.5–25 mg/kg/day for GS-441524 and 20–40 mg/kg/day for molnupiravir, depending on the presence of effusion and neurological and/or ocular signs, and continued for 84 days. Overall, 118 cats with FIP (effusive in 76) received treatment, 59 with GS-4421524 and 59 with molnupiravir. Twenty cats died, 12/59 (20.3%) in the GS-441524 group and 8/59 (13.6%) in the molnupiravir group (*p* = 0.326), with most deaths within the first 10 days of starting treatment. Among survivors, neurological and ocular signs resolved in all but one cat, who had persistent seizures. Of the cats completing treatment, 48/48 in the GS-441524 group and 51/52 in the molnupiravir group achieved remission. Laboratory parameters normalized within 6 to 7 weeks of starting drug administration. Adverse events, such as primarily hepatic function abnormalities, were transient and resolved without specific intervention. Our data indicate that GS-441524 and molnupiravir show similar effects and safety in cats with FIP.

## 1 Introduction

Feline coronaviruses (FCoV) are highly infectious enteric RNA viruses that are ubiquitous in multi-cat environments, such as boarding catteries or shelters, and are spread via the oral-fecal route (1, 2). While most FCoV infections cause minimal or no clinical signs, approximately 1 to 12% of seropositive animals are infected with virulent biotypes that cause feline infectious peritonitis (FIP), a serious and often fatal disease in cats (1, 2). FIP is a multi-system immune-related disease and a leading cause of mortality in kittens and young cats (2). There are two forms: one is a “wet” or effusive FIP, in which cats develop abdominal or pleural pockets of exudate (usually a yellow-tinged, cloudy, and mucinous fluid), and the other is a “dry” type, in which effusions are sparse or non-existent, but other clinical signs are present (2). The generalized clinical signs of FIP include malaise, poor appetite, and fever (2). In severe cases, cats may also develop ocular signs (usually uveitis) or CNS involvement, which may manifest as seizures, posterior paresis, hyperesthesia, head tilting, nystagmus, or other signs, depending on the affected site in the CNS (2).

Once cats develop overt signs of FIP, the disease is often rapidly fatal (3). Because of the serious nature of FIP, prompt treatment is indicated (4), but options have hitherto been limited (5).

Antiviral therapies have been investigated and used in cats with FIP, but none are yet approved for clinical use in Japan. GS-441524 is the active form of the prodrug remdesivir (6), a nucleoside analog used in humans. Although regulated formulations of GS-441524 are available in some countries, GS-441524 is available in Japan as an ingredient in unlicensed Mutian^®^ Xraphconn tablets or capsules (Mutian Life Sciences Co., Ltd., Nantong, China). In recent years, there has been considerable research showing that GS-441524 and remdesivir improve outcomes and reduce viral shedding in cats with FIP (7–18). However, Mutian^®^ is costly and, as an unapproved therapy, scientific information on its use in this indication is not widely available. In addition, the dose of GS-441524 in Mutian^®^ and other unregulated antiviral formulations is variable (19, 20).

Molnupiravir is another nucleoside analog used to treat severe acute respiratory syndrome coronavirus 2 and coronavirus disease (COVID-19) in humans, and has been approved in Japan since 2021 for the treatment of people with COVID-19. The availability of molnupiravir for human coronavirus infection provided an opportunity to compound this nucleoside analog as an alternative to Mutian^®^ for use in cats with FIP. At our clinic, we have been offering antiviral treatment with GS-441524 (as Mutian^®^) for cats with FIP since 2020, and in 2022, we began offering our clients molnupiravir as well, using tablets compounded at our clinic. Results of the outcomes among the first 18 molnupiravir-treated cats at our clinic have been published recently (21). This article describes outcomes in all the cats with FIP who were administered GS-441524 or molnupiravir at our clinic between June 2020 and August 2022, and were followed prospectively.

## 2 Materials and methods

### 2.1 Animals

We began offering antiviral treatment for cats with FIP from June 2020 at the You-Me Animal Clinic, Sakura City in Japan. Between June 2020 and December 2021, the only antiviral option available at our clinic was GS-441524 (Mutian^®^ Xraphconn; Mutian Life Sciences Co., Ltd., China), but, from January 2022, we began to offer molnupiravir as well. The owners were given verbal and written information about GS-441524 and molnupiravir administration before consenting to treatment, and (since January 2022) before deciding which antiviral they wanted for their cats.

This study included cats diagnosed with probable FIP who, with the informed consent of owners, began antiviral drug administration with either GS-441524 or molnupiravir between June 2020 and August 31st 2022, and were followed up for at least 11 months to August 3rd 2023. A presumptive FIP diagnosis was made using a combination of clinical signs (weight loss, reduced appetite, pyogranulomatous lesions, elevated temperature, effusions, uveitis or neurological signs), laboratory test results [presence of anemia, hyperglobulinemia, low albumin-to-globulin (A/G) ratios and high levels of α1-acid glycoprotein (α1AG)], and the identification of FCoV RNA in bodily fluid samples (22, 23). The samples (1 mL) were collected from an abdominal or pleural effusion (in cats with effusions) under ultrasound-guided abdominocentesis or thoracentesis, respectively, by fine needle aspiration (FNA) of pyogranulomatous lesions (22). Whole blood samples were collected in ethylenediaminetetraacetic acid (EDTA) tubes from each cat without effusions.

Viral detection was undertaken using reverse transcription polymerase chain reaction (RT-PCR) at the following test laboratories: abdominal effusion and FNA samples at the IDEXX Laboratory, Japan (using LightCycler^®^ 480 System II, Roche Diagnostics K.K.) or at MLT Co., Osaka, Japan (using PCR Thermal Cycler System, TaKaRa), and whole blood at the Canine Lab., Japan (using CFX Connect, Bio-Rad Laboratories, Inc.). Along with FCoV RNA analysis, effusion samples were evaluated for the total nucleated cell count, protein content, A/G ratio and cytology.

### 2.2 The drug preparation

GS-441524 was administered using commercially available Mutian^®^ Xraphconn, as capsules until June 2021 and as tablets thereafter. The molnupiravir was administered as tablets that were compounded in house at the You-Me Animal Clinic. In brief, we removed the powder from 20 commercially sourced molnupiravir 200 mg capsules [Movfor^®^, Batch No. HH2201001 (Hetero Healthcare; Hyderabad, India)], and added microcrystalline cellulose powder (Nichiga, Japan) to make a total of 12 g of powder. The powders were mixed using a mortar and pestle (Matsuyoshi Medical Instruments Co., Ltd.), and then shaped into approximately 200 tablets, each six-mm wide with a secant line, using a generic tablet press made in China (no brand name) or LFA Machines Oxford Ltd. Each tablet contained 20 mg of molnupiravir.

### 2.3 Drug administration

Antiviral drug administration was initiated when FIP was highly suspected based on the presence of clinical signs, laboratory test results and/or PCR findings as described above; the date on which treatment was started is designated as the first visit in this manuscript.

The basic dose of Mutian^®^ Xraphconn was 100 mg/kg as per manufacturer’s instruction at the time that this study began. The dose of each antiviral drug was determined by the clinical status of each cat. Based on previous studies with Mutian^®^ Xraphconn (12, 14), we prescribed 100 mg/kg for cats with effusive FIP, 150 mg/kg/day for cats with the non-effusive type and cats with pyogranulomatous lesions, and 200 mg/kg/day for cats with neurological or ocular signs of FIP. For these doses, we calculated the number of Xraphconn tablets/capsules required, and advised the owners to administer these once daily. Based on subsequent information from the manufacturer that each 100-mg capsule or tablet of Mutian^®^ Xraphconn contains 12.5 mg of GS-441524 content of each, the doses used equate to a GS-441524 dose of 12.5 mg/kg for cats with effusive FIP, 18.75 mg/kg for those with non-effusive FIP or pyogranulomatous lesions, and 25 mg/kg for cats with neurological or ocular signs.

Molnupiravir was administered at a dose of 20 mg/kg/day (as 10 mg/kg twice daily) for cats with the effusive type, 30 mg/kg/day (15 mg/kg twice daily) for cats with the non-effusive type or with pyogranulomatous lesions, and 40 mg/kg/day (20 mg/kg twice daily) for cats with neurological or ocular signs of FIP. The rationale for these doses is based on the human dose of molnupiravir and described in detail in our previous report (21). The dose could be increased or decreased in animals that showed evidence of clinical worsening or adverse events, respectively.

Owners were instructed to administer the GS-441524 tablets/capsules or molnupiravir tablets for a planned treatment duration of 84 days. For twice daily administration, owners were advised to separate doses by 12 h. The treatment was prescribed at each clinic visit, which were scheduled for 1, 2, 6 and 10 weeks after starting treatment. The decision to discontinue treatment on Day 84 or extend treatment for longer was made at the week 10 visit. If the cat did not visit the clinic during week 10, an extra 10 days of medication was given so that the cat could continue treatment until the next visit.

### 2.4 Measurements and assessments

The date of disease onset was estimated from interviews with the owners. If the owner did not recall the time when he/she noticed something wrong with their cat, we defined the date of onset as the earliest date (for example, if the owner said that it was sometime in July, we estimated the onset date as 1st of July, or if the owner said the onset was one or two weeks ago, we assumed onset was 14 days before).

We asked the owners to return for clinical follow-up 1, 2, 6 and 10 weeks after starting antiviral treatment, and between visits to record the cat’s body weight, body temperature, physical activity, appetite and defecation/urination each day. Peripheral blood samples were taken at each visit for measurement of red and white blood cell counts, hemoglobin, hematocrit (HCT), α1AG, total protein, albumin, A/G ratio, aspartate transaminase (AST), alanine transaminase (ALT), total bilirubin, creatinine, and blood urea nitrogen (BUN). The A/G ratio was determined from a fractionated protein sample. Six cats had their initial (baseline) A/G ratio measured at other clinics, and these assessments were made using biochemical methods.

Ultrasound assessments of the abdomen and chest of each cat were conducted using a Prosound α7 device (Aloka, Japan) or 65LE (Fujifilm, Japan) when treatment was initiated and after 2, 6 and 10 weeks of treatment. During the chest ultrasound, we assessed cardiac function parameters including fractional shortening, ratio of left atrial to aortic diameter, and valve regurgitation.

Owners were also asked to return for follow-up visits one and three months after completing treatment. Remission was defined as the absence of clinical signs and/or laboratory abnormalities three months after the last antiviral administration.

### 2.5 Adverse events

We recorded as adverse events any unusual laboratory test values or health events that developed during administration, and whether the dose needed to be adjusted and/or treatment discontinued.

### 2.6 Statistical analysis

Descriptive statistics were obtained for demographics and clinical variables and summarized as mean (SD) or median (range). Comparisons between the GS-441524 and molnupiravir treatment groups were performed for survival rate, as well as αA1G and A/G ratio at particular times during the treatment course (at follow up visits 1, 2 to 3, 6 to 7, 9 to 10 and 15 to 16 weeks after treatment initiation), using the Mann–Whitney U test. Fisher’s exact tests were used for categorical data. The statistical significance was defined as *p* < 0.05. Statistical analyses were conducted using R (ver. 4.2.0) and Phyton 3.10.12.

### 2.7 Ethics

All owners provided written informed consent before treatment was started. Experimental use of molnupiravir was approved by our institutional animal study review board.

## 3 Results

### 3.1 Demographics and clinical characteristics

Overall, 118 cats were treated with antivirals at our clinic between the specified dates, including the 18 described in our previous report (21); 8 (6.7%) were given a diagnosis of FIP at our clinic and the remaining 110 cats (93.2%) were given the first diagnosis at other clinics. Fifty-nine cats started treatment with GS-441524 from July 2020, with the last cat starting GS-441524 administration in July 2022. Between January 2022 and August 2022, an additional 59 cats initiated treatment with molnupiravir.

The clinical characteristics of those 118 cats at the time of treatment initiation are presented in Table 1. All 118 cats had a low serum A/G ratio, 108 had appetite loss and 84 (71.2%) had mild to severe anemia according to the HCT levels (reference interval: 30.3 to 52.3%).

**Table 1:** Baseline characteristics of the cats in this study.

	GS-441524 (*n* = 59)	Molnupiravir (*n* = 59)	*p*-value
Age at disease onset, months, median (range)	9.3 (3.3–151.1)	9.0 (2.5–181.1)	0.467^a^
Breed, *n* (%)			0.155^b^
Domestic mixed-breed	22 (37.2)	32 (54.2)	
British shorthair	3 (5.0)	4 (6.8)	
Maine Coon	3 (5.0)	4 (6.8)	
Other	31 (52.5)	19 (32.2)	
Sex, *n* (%)			0.765^b^
Male intact/male neutered	18/23	19/19	
Female intact/female neutered	8/10	7/14	
Weight, kg, mean (SD)	2.6 (1.04)	2.9 (1.03)	0.157^a^
Duration from disease onset to treatment initiation^c^, days, median (range)	8 (1–30)	12 (1–210)	0.025^a^
Effusive type, *n* (%)	42 (71.1)	36 (61.0)	0.331^b^
Pyogranulomatous lesion in abdomen, *n* (%)	11 (18.6)	22 (37.2)	0.039^b^
Neurological or ocular signs of FIP, *n* (%)	16 (27.1)	20 (33.9)	0.549^b^
Temperature, °C, mean (SD)	39.5 (0.7)	39.4 (0.84)	0.318^a^
Hematocrit, %, mean (SD)	24.8 (8.2)	26.8 (7.8)	0.240^a^
Anemia^d^, *n* (%)	42 (71.2)	42 (71.2)	
Albumin/globulin ratio, mean (SD)	0.38 (0.12)	0.36 (0.11)	0.278^a^
Sample type for FCoV RT-PCR, *n* (%)			0.098^b^
Abdominal effusion	34 (57.6)	26 (44.1)	
Pleural effusion	5 (8.5)	9 (15.3)	
FNA of pyogranulomatous lesion	7 (11.9)	12 (20.3)	
Whole blood	9 (15.3)	12 (20.3)	
None	4 (6.8)	0	

FCoV, feline coronavirus; FIP, feline infectious peritonitis; FNA, fine needle aspiration; RT-PCR, real-time polymerase chain reaction; SD, standard deviation.

a Mann-Whitney U test.

b Fisher’s exact test.

c Based on owner’s report of when signs of illness first appeared.

d Hematocrit below the reference interval of 30.3 to 52.3%.

In the GS-441524 group, 42 (71.2%) cats had effusive FIP and the remaining 17 cats had non-effusive FIP. Pyogranulomatous lesions were found in the abdominal cavity in 11 cats, and 16 (27.1%) showed neurological or ocular signs. In the molnupiravir treatment group, FIP was effusive in 36 (61.0%) and non-effusive in 23, pyogranulomatous lesions were found in the abdominal cavity in 22 cats, and 20 (33.9%) had neurological or ocular signs of FIP.

### 3.2 Administration and dose

Twenty-nine of the cats in the GS-441524 group were treated between July 2020 and December 2021 (when that was the only available treatment), and 30 received GS-441524 during the period when molnupiravir was also available.

The median duration from the estimated date of disease onset to initiation of antivirals was 10 (range 1–210) days.

Overall, 48 (81.3%) cats completed 84 days of administration with GS-441524, and 52 (88.1%) with molnupiravir.

Five cats received extended administration of GS-441524 because they did not attend the week 10 check-up at the clinic (n = 2), the A/G did not return to normal (n = 2), or the cat (n = 1) had persistent neurological signs (wobble). Three cats extended administration of molnupiravir, because the A/G had not fully normalized at week 10. A summary of treatment administration is shown in Table 2.

**Table 2:** Summary of outcomes of in cats who completed treatment.

	GS-441524 (*n* = 48)	Molnupiravir (*n* = 52)	*p*-value
Outcomes, *n* (%)			1.000^a^
Remission	48 (100.0)	51 (98.0)	
Death within 3 months of completion	0	1 (1.9)	
Death after 3 months of completion	1*	0	
Total days of administration, median (range)	84 (84–192)	84 (84–100)	0.579^b^
Values at the final treatment visit, mean (SD)			
Weight, kg	3.36 (0.803)	3.56 (0.862)	0.265^b^
Temperature, °C	38.37 (0.234)	38.25 (0.366)	0.237^b^
Hematocrit, %	39.63 (7.71)	40.42 (8.27)	0.651^b^
Albumin/globulin ratio	0.71 (0.132)	0.69 (0.141)	0.359^b^

*One cat, who had achieved remission and remained healthy at the 3-month post-treatment visit, later died from anemia 114 days after treatment completion.

a Fisher’s exact text.

b Mann-Whitney U test.

### 3.3 Outcomes

Eleven cats (18.6%) in the GS-441524 group and seven (11.96%) in the molnupiravir group died during antiviral administration, and one in each group died after completing treatment, for a total death rate of 12/59 (20.3%) in the GS-441524 group and 8/59 (13.6%) in the molnupiravir group. Most of these deaths (10/12 [83.3%] in the GS-441524 group and 5/8 [62.5%] in the molnupiravir group) occurred within 10 days of starting treatment, and five in the GS-441524 group and one in the molnupiravir group occurred one day after treatment initiation.

Two cats in the molnupiravir group died 56 and 67 days after starting antiviral administration, respectively; the cause of both deaths was heart failure secondary to hypertrophic cardiomyopathy. One cat receiving GS-441524 died 24 days after treatment initiation and the owner did not share the cause of death with us.

Overall, 48 cats receiving GS-441524 (81.3%) and 52 receiving molnupiravir (84.7%) completed treatment and among them, 48/48 (100.0%) of GS-441524-treated and 51/52 (98.1%) of molnupiravir-treated cats achieved remission [odds ratio 0.941 (95%) confidence interval 0.572–15.473; *p* = 1.0]. The outcomes of cats who completed treatment are summarized in Table 2. One cat in the molnupiravir group did not achieve remission and died 27 days after treatment completion (111 days after initiation). The cat was positive for feline leukemia virus and died due to acute myeloid leukemia. Another late death occurred in a GS-441524-treated cat who had previously achieved remission. This cat showed no relapse at a clinic visit 3 months after completing the course of antivirals, but we later received a report of the death, which occurred 114 days after treatment completion (198 days after initiation), from the cat’s usual veterinarian. The cause of death was anemia. The cat had received no additional treatments after completing GS-441524.

**Figure 1:**
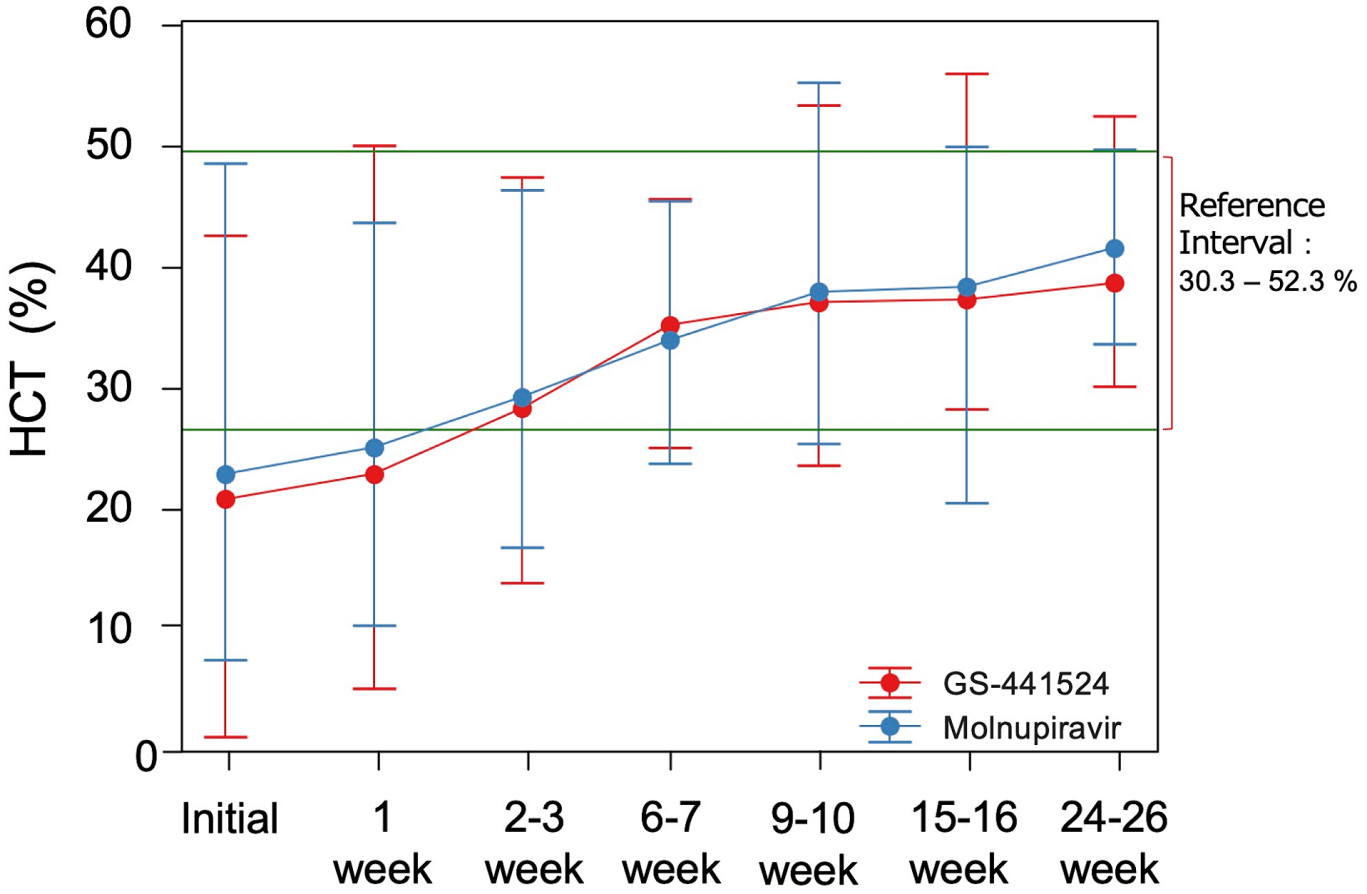
Mean (SD) hematocrit (HCT) levels over time. Normal range is 30.3–52.3%.

**Figure 2:**
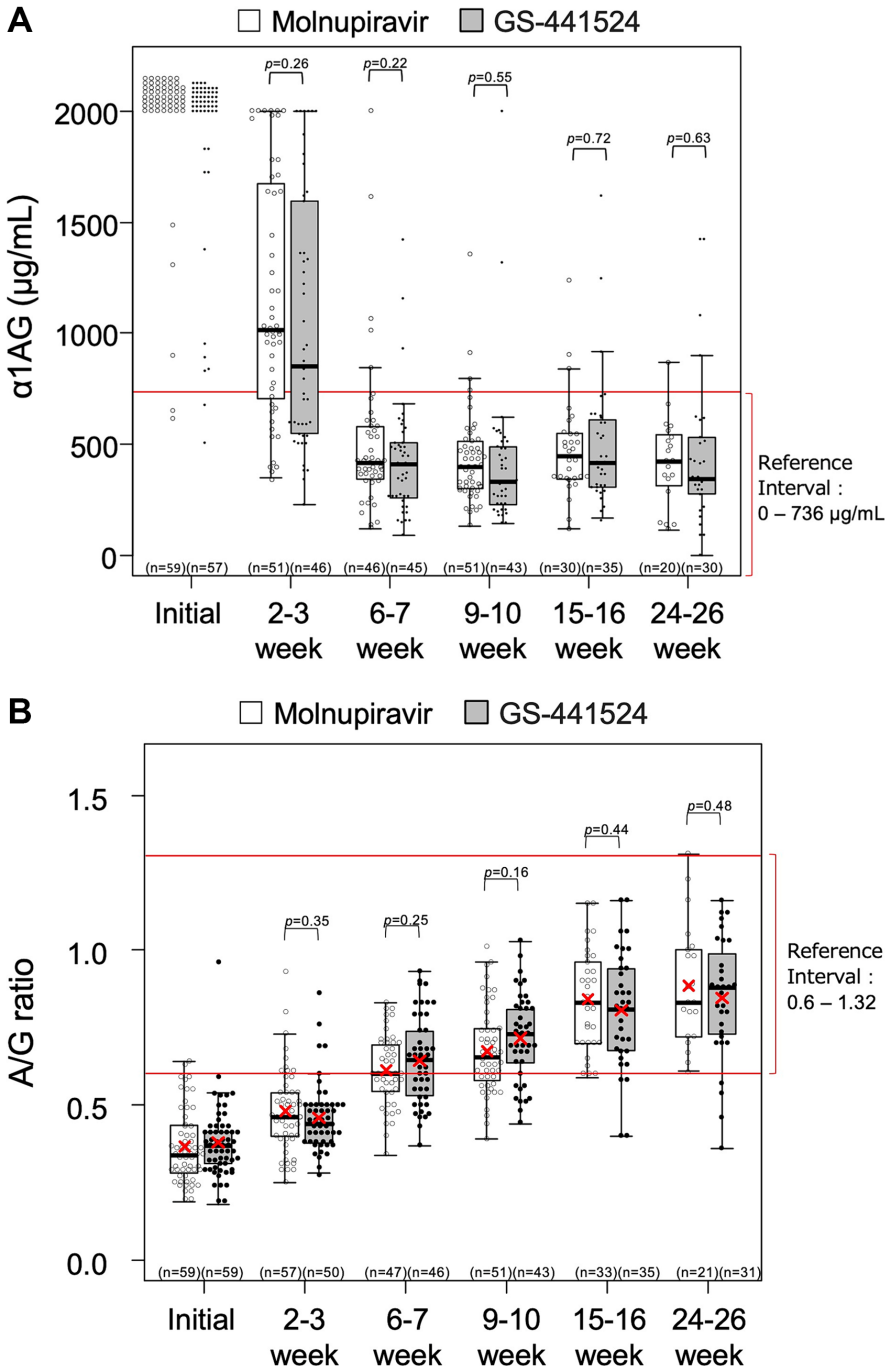
Levels of **(A)** α1-acid glycoprotein (α1AG) and **(B)** albumin-to-globulin (A/G) ratio over time. Each rectangle represents the interquartile range and the horizontal line is the median. Vertical lines indicate the statistical range where the minimum is calculated as Q1–1.5*IQR and the maximum is calculated as Q3 + 1.5*IQR, where IQR is interquartile range. The red X indicates mean, where calculated. Descriptive statistics for α1AG level at week 0 were not calculated because most of the values were > 2000 μg/mL.

The changes in HCT values, α1AG and A/G ratio for all cats are shown in Figures 1, 2. There were no statistical differences between the two groups in these parameters. Among the 99 cats who achieved remission, body temperature had returned to normal at the week 1 follow-up visit. The HCT values had returned to the reference interval 6–7 weeks after starting antiviral treatment (Figure 1), as had α1AG and A/G ratio values (Figures 2A,B).

As of August 3 2023, 47 cats in the GS-441524 group remained alive 341 to 1,043 days after completing treatment, and 51 cats in the molnupiravir group remained alive 261 to 472 days after completing treatment.

### 3.4 Neurological or ocular signs

Neurological or ocular signs were present at the first visit to our clinic in 16 (27.1%) cats receiving GS-441524 and in 20 (33.9%) receiving molnupiravir. Of the 16 cats in the GS-441524 group, five cats died and one cat had persistent seizures that had not resolved by Day 134, so administration was discontinued. As of August 2023, this cat was alive and seizures were controlled by phenobarbital administration. Neurological signs resolved within 7 to 43 days (median 8 days) in the other 10 cats. All neurological or ocular signs resolved within 3 to 43 (median 7) days in the 20 cats receiving molnupiravir.

One cat in the GS-441524 group and three in the molnupiravir group developed neurological signs during administration, on day 15 (in the cat receiving GS-441524 and one receiving molnupiravir) or day 7 (in two cats receiving molnupiravir). In response to these neurological signs, the dose of GS-441524 was increased from 5 to 10 mg/kg/day on Day 15, and the dose of molnupiravir increased from 20 to 40 mg/kg/day on Day 7 in one cat and day 15 in another, and from 30 to 40 mg/kg/day on Day 7 in the third. All neurological signs had resolved by the next visit after the dose increase, which was one week later in the cats having a dose increase on day 7, and four weeks later in the cats having a dose increase on day 15.

### 3.5 Safety

An increase of ALT values was observed during treatment in 20 cats receiving GS-441524 and 18 receiving molnupiravir. These ALT abnormalities were detected 4 to 75 (median 8) days after initiation of GS-441524, and 5–71 (median 8) days after initiation of molnupiravir.

Abnormalities in total bilirubin were observed in two cats treated with GS-441524 and three cats with molnupiravir, and abnormalities in creatinine concentrations were observed in one cat receiving molnupiravir (peak level of 2.9 mg/dL) but none receiving GS-441524. No clinical signs of renal disease, such as polydipsia, polyuria or anuria, were observed.

Two cats receiving GS-441524, but none receiving molnupiravir, developed folded ears, but these returned to normal 69 and 45 days after antiviral discontinuation, respectively. Five (two receiving GS-441524 and three receiving molnupiravir) also developed hair loss; the hair loss was patchy and localised to the lower jaw and behind the ears in the two cats receiving GS-441524 and in one cat receiving molnupiravir, but manifested as hair thinning all over the bodies of two cats receiving molnupiravir. All affected cats had confirmed new hair growth within 14 to 88 (median 40) days of treatment discontinuation, without further management. No severe adverse events were found in either group.

### 3.6 Follow-up

Of the 99 cats that achieved remission, no relapses had occurred in 98 (99.9%) by 3^rd^ August, 2023. One cat died 114 days after administration completion as described above (section 3.3 Outcomes). Overall, 47 cats treated with GS-441524 were in good health and condition 114 to 1,043 days after treatment completion, and 50 cats treated with molnupiravir were in good health/condition 27 to 472 days after finishing the antiviral course.

## 4 Discussion

Here, we describe outcomes in all cats receiving antiviral treatment for FIP at our clinic between July 2020 and August 2023, including the first 18 cats treated with molnupiravir reported previously (21). Our aim was to determine whether there are differences between GS-441524 and molnupiravir in terms of effectiveness and safety, but we noted no statistically significant differences in survival rate, timing of clinical improvement or adverse event incidence. High remission rates were achieved in both treatment groups, and important signs of illness (including body temperature, HCT, α1AG and A/G ratio values) had returned to normal within 7 weeks of starting either treatment.

Most of the 118 cats involved in this study received the diagnosis of FIP at other clinics and/or hospitals. Owners of those cats visited our clinic after researching possibilities for treatment, which we offered in the form of GS-441524 between July 2020 and January 2022, and as a choice between GS-441524 and molnupiravir from January 2022 onwards. Among those offered a choice of administration, 30 owners chose GS-441524 and 59 chose molnupiravir. Since the data cutoff for the current analysis (August 2022), 22 clients have chosen molnupiravir and one has chosen GS-441524, suggesting that molnupiravir is the preferred choice at our clinic. We have not formally investigated the reasons for this, but suspect it is related to the lower price of molnupiravir, and to our ability to demonstrate good results with this agent (21).

In our cohort of cats with FIP, 20 died after starting antiviral treatment: 12 in the group receiving GS-441524 (20.3%) and eight in the group receiving molnupiravir (13.6%). This is consistent with previous findings, which report a mortality rate during GS-441524 or remdesivir of between 0 and 44% (8–14, 16, 17). The two largest studies with GS-441524 or remdesivir (each in more than 300 cats) report a mortality rate of around 11% [10.5% in one (13) and 11.4% in the other (17)]. Comparatively less mortality data were available on the impact of molnupiravir. In our previous case series, reporting results from the first 18 cats receiving molnupiravir at our clinic, the mortality rate was 22.2% (4/18 cats) (21). In a separate report by Roy and colleagues, the mortality rate was 7.7% (2/26 cats), but all of these animals had previously received GS-441524 or remdesivir, and were given molnupiravir for relapsed or persistent clinical signs of FIP (24), so the cohort was not clinically comparable to the cats in our study.

In our cohort, 10 deaths (50%) occurred within the first four days of administration. This is consistent with previous reports in cats receiving antiviral therapy, which commonly show the highest rate of deaths within the first few days of starting administration (8–10, 12–14, 16, 17, 21). Because antiviral treatment was initiated 3 to 14 (median 6.5) days after disease onset, we can assume that the deaths were due to acute and severe disease progression. Of the 10 early deaths in our cohort, eight cats received GS-441524 and two were cats received molnupiravir. We speculate that there were no specific reasons for the difference.

No safety concerns requiring special attention were noted in either group. The most common adverse event during antiviral administration in our cohort was an increase in ALT values, which was observed in 20 cats receiving GS-441524 (33.9%) and 18 receiving molnupiravir (30.5%); however, all were temporary and resolved without management. Transient and self-limiting increases in ALT have been noted previously in cats receiving GS-441524 (8, 10, 14, 17), although other researchers have reported no change in hepatic enzyme levels during GS-441524 administration (9, 16). Other biochemical abnormalities noted in our animals, such as increases in creatinine concentration and total bilirubin, have also been reported during GS-441524 treatment (10, 17). All biochemical and clinical abnormalities (including hair loss and folded ears) in our cohort of cats receiving nucleoside analogs resolved without intervention.

It is interesting to note that two cats receiving GS-441524 in our study developed folded ears, because, to the best of our knowledge, this side effect has only been reported once before in a cat receiving antiviral treatment for FIP (24). In that case, the cat developed folded ears while receiving molnupiravir, but this cat had received two courses of injectable GS-441524 prior to receiving molnupiravir, and was receiving the highest dose of molnupiravir (30 mg/kg twice daily) when the folded ear tips developed (24). Other large-scale studies with GS-441524 do not report any cases of folded ears (12–14, 17). It is possible that the development of folded ears is a side effect of a component of Mutian^®^ Xraphconn other than GS-441524. The potential relationship between antiviral treatments and ear folding warrants further investigation.

We believe that this is the first study to compare the effects of GS-441524 and molnupiravir in cats with FIP. However, our study is not without limitations. First, this was not a preplanned prospective study, so no randomization or blinding was employed, which may have contributed to bias. We began offering GS-441524 as a treatment for FIP in January 2020 and molnupiravir in January 2022, and owners were able to choose the antiviral for their animal once molnupiravir became available. We believe that bias in the allocation of treatment was limited since all cats with presumptive FIP were offered antiviral treatment during the period of the study. However, we noted that, in recent months, an increasing number of owners chose molnupiravir, the less expensive option.

Neither of the antiviral agents offered at our clinic are registered veterinary medicines. Therefore, the optimal dosages of these treatments are unknown. At the time we began offering molnupiravir to our clients, there were no available data on the optimal dosage to use in cats, and we based our dosage estimates on internet sources that suggested a minimum effective oral dose of molnupiravir of 9 mg/kg/day for cats with no neurological or ocular signs and up to 50 mg/kg/day for those with neurological signs (25, 26). Our dose calculations also assumed that the pharmacokinetic profile of molnupiravir in cats would be comparable to that in humans (21). Since then, data on the pharmacokinetics of molnupiravir in cats have been published (27), and support the choice of dose in our study. Molnupiravir is rapidly absorbed in cats after oral administration (27). Serum levels of the active metabolite (NHC) reach and exceed the 50% maximally effective concentration (EC_50_) soon after oral administration of 10 mg/kg, and the mean time to maximal serum concentration is 4.3 h (27). In this pharmacokinetic study, molnupiravir was administered in gelatine capsules containing powder without excipients (27), whereas we compounded tablets with microcrystalline cellulose. However, we have no reason to consider that the addition of this excipient would have a clinically significant effect on the pharmacokinetics of molnupiravir (28).

The optimal dose of GS-441524 for cats with FIP has not been established. Previous studies have used a range of doses from 2 to 27 mg/kg (8, 9, 12–17), most commonly ~10 mg/kg (8, 10, 13–15, 17). The doses used in the current study are at the higher end of this dose range (12.5 to 25 mg/kg). We may study the efficacy and safety of lower doses of GS-441524 in the future.

Another potential issue with unregulated medicines is that there may be inconsistency in the quality and dose of nucleoside analog in each tablet. This has been noted before in commercially available GS-441524 preparations (18, 20). A recent US report noted that 43% of the popular commercial oral formulations containing GS-441524 include a higher than expected dose (an average of 75% above expected levels), and 58% contain a lower than expected dose (an average of 39% less than expected) (19). We did not test the treatments to determine the concentration of active substance, so we cannot rule out the possibility of some inconsistencies. Nor can we rule out the possibility that ingredients in Mutian^®^ Xraphconn other than GS-441524 may have influenced clinical outcomes.

Finally, our study was limited to domestic cats kept as pets, and did not include animals from shelters or breeding facilities. Nor did we evaluate the FIP serotype or genotype, so it is not possible to determine whether the results are specific to particular FCoV biotypes or variants.

In conclusion, the current study complements our previous report (21) in providing information on the effectiveness and safety of molnupiravir in cats with FIP, and demonstrates that GS-441524 and molnupiravir are similarly effective and safe in this condition. Owners of cats with FIP can be advised that the disease is curable and there is more than one antiviral option available. We would encourage the development of nucleoside analogs as registered veterinary medicines for FIP to ensure the quality and consistency of treatment, and to optimize the dosing of these treatments in cats.

## Data availability statement

The raw data supporting the conclusions of this article will be made available by the authors, without undue reservation.

## Ethics statement

Experimental use of molnupiravir was approved by the Institutional Review Board of the You-Me Clinic.

## Author contributions

OS: Conceptualization, Data curation, Formal analysis, Funding acquisition, Investigation, Methodology, Project administration, Resources, Supervision, Validation, Visualization, Writing – original draft, Writing – review & editing. TI: Data curation, Formal analysis, Investigation, Writing – review & editing. TakS: Data curation, Formal analysis, Investigation, Writing – review & editing. TadS: Supervision, Writing – review & editing.
